# Genome-wide analysis of WRKY transcription factor genes in *Toona sinensis*: An insight into evolutionary characteristics and terpene synthesis

**DOI:** 10.3389/fpls.2022.1063850

**Published:** 2023-01-20

**Authors:** Liping Ren, Wenyang Wan, Dandan Yin, Xianhui Deng, Zongxin Ma, Ting Gao, Xiaohan Cao

**Affiliations:** ^1^ Key Laboratory of Horticultural Plant Biology of Biological and Food Engineering School, Fuyang Normal University, Fuyang, China; ^2^ Horticultural Institute, Fuyang Academy of Agricultural Sciences, Fuyang, China; ^3^ State Key Laboratory of Tea Plant Biology and Utilization, International Joint Laboratory on Tea Chemistry and Health Effects, Anhui Agricultural University, Hefei, China

**Keywords:** *Toona sinensis*, *WRKY* transcription factors, gene evolution, terpene synthesis, genome-wide analysis

## Abstract

*WRKY* transcription factors (TFs), one of the largest TF families, serve critical roles in the regulation of secondary metabolite production. However, little is known about the expression pattern of *WRKY* genes during the germination and maturation processes of *Toona sinensis* buds. In the present study, the new assembly of the *T. sinensis* genome was used for the identification of 78 *TsWRKY* genes, including gene structures, phylogenetic features, chromosomal locations, conserved protein domains, cis-regulatory elements, synteny, and expression profiles. Gene duplication analysis revealed that gene tandem and segmental duplication events drove the expansion of the *TsWRKYs* family, with the latter playing a key role in the creation of new *TsWRKY* genes. The synteny and evolutionary constraint analyses of the *WRKY* proteins among *T. sinensis* and several distinct species provided more detailed evidence of gene evolution for *TsWRKYs*. Besides, the expression patterns and co-expression network analysis show *TsWRKYs* may multi-genes co-participate in regulating terpenoid biosynthesis. The findings revealed that *TsWRKYs* potentially play a regulatory role in secondary metabolite synthesis, forming the basis for further functional characterization of *WRKY* genes with the intention of improving *T. sinensis*.

## 1 Introduction

*Toona sinensis* (A. Juss) Roem, a deciduous native plant endemic to eastern and southeastern Asia and commonly known as Chinese toon, belongs to the Meliaceae family (Dong et al., 2013). In China, the tender buds of *T. sinensis* have been accepted widely as vegetables for its rich nutritional value and unique aroma (Zhai and Granvogl, 2019). The young leaves of *T. sinensis* are high in amino acids, vitamins, and other nutrients that are beneficial to human health (Ren et al., 2021). *T. sinensis* is often known as traditional Chinese medicine due to the use of its numerous tissues in the treatment of various of ailments. A recent study showed that terpenoids, phenylpropanoids, and flavonoids, known as bioactive substances derived from the extracts of *T. sinensis* leaves and bark have been identified to have anti-tumor, antioxidant, anti-inflammatory, antibacterial, antiviral, hepatoprotective, and hypoglycemic effects (Ji et al., 2021). *T. sinensis* in Taihe, Anhui has many varieties such as ‘Heiyouchun’, the most famous variety because of its taste, aroma, and nutritional value that was offered as a tribute as early as the Tang Dynasty (Yang et al., 2020a). In the early stage when the solar term of Grain Rain is coming, the ‘Heiyouchun’ shows the best quality, strong aroma, and good taste because its sprouts are thick, fat, and tender with the best oils, strong fragrances, and crunchiness (Sui et al., 2019).

*WRKYs* are plant-specific transcription factors (TFs) and have also been found in protozoans (*Giardia lamblia*) and amoeboid (*Dictyostelium discoideum*), indicating a long evolutionary history (Goyal et al., 2020; Mao et al., 2020). The DNA-binding domain of WRKY TFs is 60 amino acids long and has a highly conserved heptapeptide (WRKYGQK) signature motif on the N-terminus and a zinc finger-like motif on the C-terminus (Eulgem et al., 2000). This domain forms a four-stranded β-sheet whose stability is determined by a zinc-binding pocket at the end of the β-sheet, suggesting that the N-terminal conserved sequence can bind directly to DNA (Rushton et al., 2010; Rinerson et al., 2015). *WRKYs* are classified into three categories (Groups I–III) based on the number of conserved domains and the type of zinc finger structure. The first type (Group I) has a WRKY domain at the C-terminal and N-terminal, whereas the second type (Group II) also has a WRKY domain, and both types are C2H2 type zinc finger structures. The third type (Group III) is constituted of a single WRKY domain with the zinc finger structure of the C2HC type. Further, group II proteins could be classified into five primary subgroups (IIa+b, IIc, IId+e), depending on the evolutionary relationship of the WRKY domains (Eulgem et al., 2000; Agarwal et al., 2011).

Sweet Potato Factor 1 (SPF1), the first WRKY cDNA-encoding DNA-binding protein, was discovered in the 5’ upstream region of three genes associated with the synthesis of sporamin and amylase in sweet potato tuberous roots (*Ipomoea batatas* L.**)** (Ishiguro and Nakamura, 1994). Following that, *WRKYs* are found across the genome and in a multitude of crop species, including cotton (*Gossypium hirsutum* L.) (Dou et al., 2014), mouse-ear cress (*Arabidopsis thaliana* L.) (Wang et al., 2011), rice (*Oryza sativa* L.) (Ross et al., 2007), and sesame (*Sesamum indicum* L.) (Li et al., 2017), Banana (*Musa acuminata*) (Jia et al., 2022). Significant evidence shows that *WRKYs* are required for a variety of physiological processes, including embryogenesis (Yang et al., 2020b), seed dormancy and germination (Zhou et al., 2020), trichome initiation (Xie et al., 2021), root growth (Rosado et al., 2022), blooming time (Li et al., 2016), fruit ripening (Cheng et al., 2016), senescence (Fan et al., 2017), and metabolic activities (Schluttenhofer and Yuan, 2015). Additionally, *WRKYs* also act as both positive and negative regulators of plants’ responses to biotic and abiotic stresses (Song et al., 2014). It is worth noting that the regulatory activity of *WRKYs* is associated with several signaling pathways, including jasmonic acid, salicylic acid, and abscisic acid, all of which are related to abiotic stress responses (Chen et al., 2010; Dang et al., 2013; Yan et al., 2014).

Furthermore, it is improbable that the role of *WRKYs* will be confined to coordinated defensive reactions. The *WRKYs* regulate the biosynthetic genes involved in terpenoid synthesis by activating or inhibiting transcription, either alone or in combination with other TFs (Schluttenhofer and Yuan, 2015). *GaWRKY1* from cotton has been demonstrated to bind specifically to the W-box in the CAD1-A promoter and control the activity of the cotton *CAD1* gene, implicating a role in sesquiterpene biosynthesis regulation (Xu et al., 2004). When methyl jasmonate induces *Medicago truncatula*, several *WRKY* genes involved in the production of defensive chemicals (terpenoids and isoflavonoids) are upregulated (Naoumkina et al., 2008). In periwinkle(*Catharanthus roseus*), the *CrWRKY1* gene is selectively expressed in roots, following JA and ethylene exposure, and it interacts with the *DXS* and *SLS* genes involved in steroid production, as well as with the regulators *CrMYC2* and *CrZCT* (Suttipanta et al., 2011). These combined results provide insights on the terpene synthase mechanism of the WRKY gene family in plants. Terpenoids are versatile natural compounds that act as metabolic mediators, ecological communicators, and plant volatiles. As vegetables, terpenes, in addition to their major contribution to the taste of the plant, also have pharmacological effects: anti-cancer, anti-viral, and cholesterol-lowering. Recently, a study discovered 109 chemicals in *T. sinensis* tissues, including terpenoids, phenylpropanes, and flavonoids (Peng et al., 2019). Since the biosynthesis of terpene compounds is usually mediated by the terpene synthase (*TPS*) family, it is difficult to significantly increase the content of specific terpenoids through the regulation of a single enzyme gene. *WRKY* gene family regulate the secondary metabolism of various plants, especially the enzymes associated with terpenes biosynthesis. In particular, the *WRKY* gene family in *T. sinensis* has not been fully described, and the roles of the genes within the species remain unknown. Therefore, it is critical to identify and fully investigate the *WRKY* gene family related to terpene biosynthesis in *T. sinensis*.

As one of the best-known vegetables, few terpenoid-relative genes have been identified, and the molecular genetic basis of terpenoid biosynthesis pathways is still unveiled. This work found 78 members of the *TsWRKYs* genes family and determined their biochemical properties, phylogeny, gene structure, conserved motifs, gene promoters, chromosomal distribution, and evolution processes. In addition, *TsWRKYs* and terpenoid synthase gene expression patterns were analyzed across different young leaf sampling periods. Our study comprehensively revealed the information of the *TsWRKYs*, which is beneficial to promoting the discovery of its regulatory network and unique function in regulating the synthesis of volatile aromatic compounds.

## 2 Materials and methods

### 2.1 Identification of the *WRKY* genes family members in *T. sinensis*

The complete genome and proteome sequences of *Arabidopsis* were downloaded from the Arabidopsis Information Resource^1^. The *T. sinensis* data reported in this study are available under Accession No. CNP0000958 in the CNGB Nucleotide Sequence Archive^2^. The hidden Markov model (HMM) file of the WRKY domain (Accession Number PF03106) was downloaded from the Pfam database^3^ (Mistry et al., 2021), and HMMER3.0 was used for the identification of *WRKY* genes with an E-value setting of 1e-5. In addition, SMARAT^4^ and CCD^5^ were used to confirm all the potential *TsWRKY* genes (Chen et al., 2018; Lu et al., 2020; Letunic et al., 2021). The molecular weights (Mw), instability index (II), aliphatic index (AI), the Grand Average of Hydropathicity (GRAVY), and isoelectric points (pI) of the identified WRKY proteins were predicated on the Expasy website^6^ (Gasteiger et al., 2005). The subcellular locations were predicted using ProtComp – Version 9 from Softberry website ^7^.

### 2.2 Sequence analysis and Cis-regulatory element prediction of *TsWRKYs* genes

Multiple sequence alignments were created using ClustalW using default settings (Thompson et al., 2003), and then the WRKY proteins conserved domain sequences were modified in GeneDoc software (Nicholas, 1997). The distribution pattern of intron was analyzed by the Gene Structure Display Server (GSDS) (Hu et al., 2015)^8^. Conserved motif analysis of the identified *T. sinensis* WRKY proteins was carried out on the MEME online program (Bailey et al., 2015)^9^. The optimized parameters of MEME are as follows: the maximum number of motifs is 20, the motif width is between 8 and 50 aa, and the rest of the parameters are default. The promoters, which were extracted from 2000 bp upstream of the CDS region of *TsWRKYs*, were used for Cis-regulatory elements (CREs) prediction analysis by PlantCARE online software (Lescot et al., 2002)^10^.

### 2.3 Chromosomal distribution and gene duplication of *TsWRKYs* genes

The Circos (Krzywinski et al., 2009) was used to map the *TsWRKY* gene information based on *T. sinensis* genomic data. Tandem and segmental duplication of *T. sinensis WRKY* genes, as well as the synteny relationship between *T. sinensis* and six plant species genomes, were evaluated using MCScanX (Wang et al., 2012) and TBtools (Chen et al., 2020). The KaKs Calculator 2.0 (Wang et al., 2010) was used to estimate the non-synonymous (Ka) and synonymous (Ks) substitution of each duplicated *WRKY* gene. The sequence of WRKY proteins from Arabidopsis, tomato, citrus, maple, pineapple, and rice was downloaded from the NCBI^11^.

### 2.4 Phylogenetic analysis and classification of *TsWRKYs* genes

The conserved domains from the predicted WRKY proteins sequences were confirmed using multiple sequence alignments. The amino acid sequences of WRKY proteins in *A. thaliana* and *T. sinensis* were alignment by ClustalW. The phylogenetic trees were constructed using the Neighbor-Joining (NJ) method in MEGA 7.0 (Kumar et al., 2016) with the following pa-rameters: p-distance, pairwise deletion, and 1000 bootstrap replications. The neighbor-joining tree construction method of *T. sinensis* and other green line species refers to the research of Rinerson (Rinerson et al., 2015). The green line species including: *Micromonas pusilla*, *Ostreococcus tauri*, *Ostreococcus lucimarinus*, *Dunaliella salina*, *Chlamydomonas reinhardtii*, *Gonium pectorale*, *Volvox carteri*, *Physcomitrella patens*, *Selaginella moellendorffii*, *Brachypodium distachyon*, *Oryza sativa*, *Glycine max*, *Arabidopsis thaliana*, and *T. sinensis*.

### 2.5 Plant materials and gene expression analysis

The *T. sinensis* var. ‘Heiyouchun’ used in this study is universally recognized as the best variety because of its nutritional value, good taste, and unique aroma. ‘Heiyouchun’ was grown in the field at the Forestry Nursery of the Taihe County, Fuyang City, Anhui Province, China (118°48’8’’ N and 32°3’52’’ E). Young and healthy leaves with at least six branches and 5–10 cm in length were collected in four different sampling periods from March 30 to April 20, 2021. All collected samples were immediately frozen in liquid nitrogen and stored at −80°C.

Total RNA from samples was extracted using the RNA Extraction Kit 3.0 (Huayueyang Biotech, Beijing, China), with the RNase-free DNase I treatment to remove potential genomic DNA contamination. Qualified RNA was chosen as a template to produce the first-strand cDNA, as determined by gel electrophoresis and the A260/A280 ratio. Complementary cDNA was generated with SuperScript cDNA Synthesis Kit WX2050 (Huayueyang Biotech, Beijing, China). The specific *TsWRKY* gene primers were designed using Primer Premier 5, and *TsActin* gene served as the reference gene for normalization of the expression levels in different sampling periods. **Supplementary Table S1** presents all the primer information. The qRT-PCR was performed with a 2×SYBR Green qPCR Mix (With ROX) (Sparkjade, Shandong, China), and amplification was performed using 96-well plates and CFX96 TouchTM RT-PCR system (Biorad, Los Angeles, CA, USA). Each reaction was performed in biological triplicates. The data from qRT-PCR amplification were analyzed using the 2^−ΔΔCt^ method. A calculation of Bonferroni’s multiple comparisons test was performed using SPSS statistical software version 25. A mean fold change greater than 2 and a p value less than 0.05 were considered significant differences between the two groups.

## 3 Results

### 3.1 Identification of the WRKY proteins in *T. sinensis*

To thoroughly investigate the candidate *WRKY* genes in *T. sinensis*, 78 *TsWRKY* genes were finally identified, designated as *TsWRKY1*–*TsWRKY78* based on the order of their HMM (Hidden Markov Model) search results, and were used for subsequent analysis. All extensive information *TsWRKYs*, including chromosomal location, subcellular localization prediction, protein length, molecular weight, GRAVY, instability index, and aliphatic index are provided on **Table S2**. Among the 78 TsWRKY proteins, TsWRKY14 and TsWRKY39 proteins were determined to be the smallest and the largest proteins, with 116 and 1161 amino acids (aa), respectively. The proteins’ mo-lecular weights varied from 13.5 to 125.8 kDa, and their pI values were from 4.94 to 9.73. According to the expected subcellular localization results, 67 and 11 TsWRKY proteins were found in the nucleus and extracellular areas, respectively. All of the TsWRKY proteins have a GRAVY of less than 0, which means that they are all hydrophilic proteins, and more information is shown in **Table S2**.

### 3.2 Multiple sequence alignment, phylogenetic analysis, and classification of *TsWRKYs* genes

**Figure 1:**
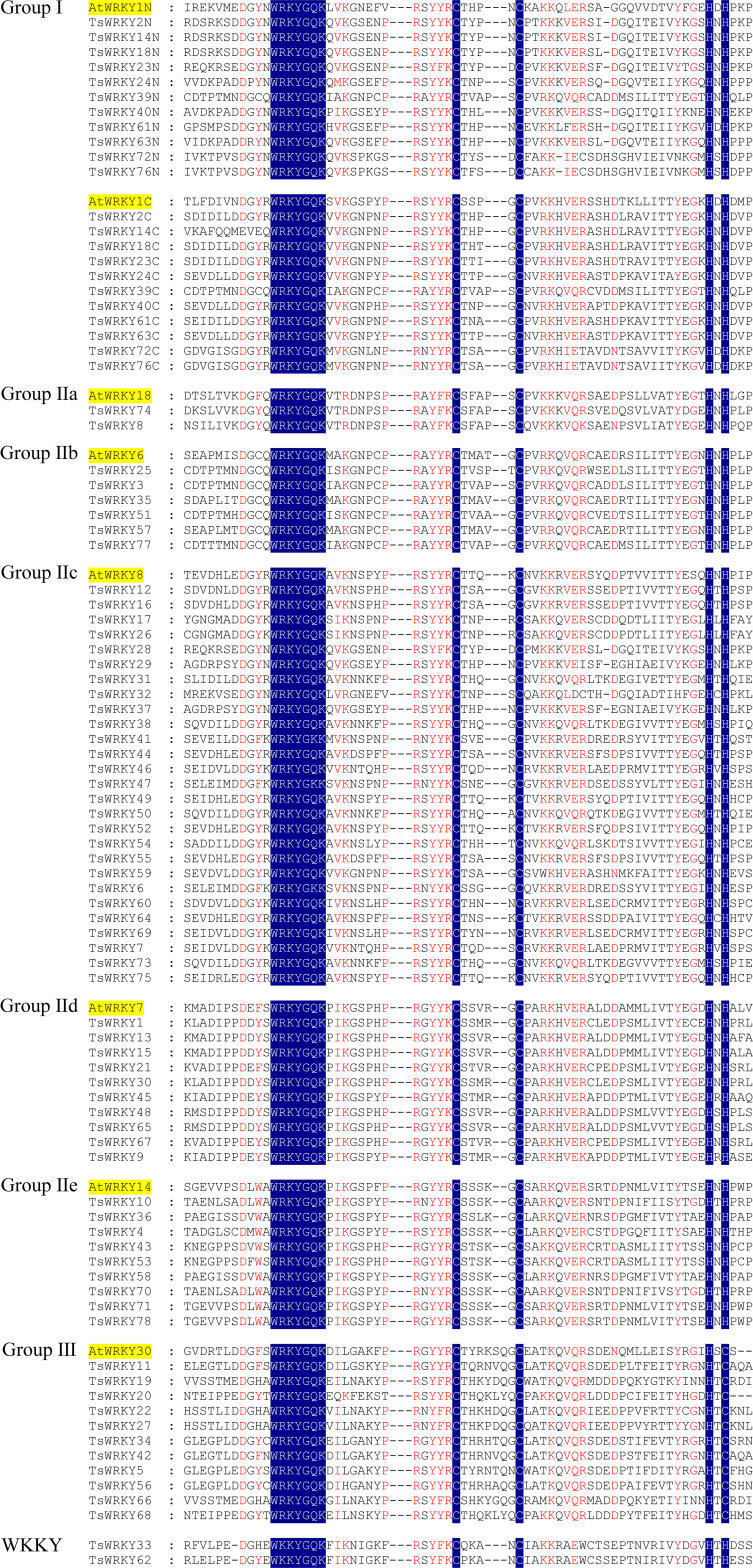
Multiple TsWRKY domain amino acid sequences were aligned with reference to AtWRKY. The letters “N” and “C” represent WRKY domains, which are found at the N and C terminal ends of amino acid sequences, respectively.

Multiple sequence alignments of the WRKY domains, which cover about 60 amino acids, were used to analyze the evolutionary relationships of TsWRKY proteins. The WRKY domains of seven distinct *Arabidopsis* WRKY proteins (*AtWRKY*1, 18, 6, 8, 7, 14, and 30) were randomly chosen as representatives for further evaluation. **Figure 1** shows the substantially conserved WRKY domain sequences. The majority of the proteins in this family (76 out of 78) share the conserved WRKY domain WRKYGQK, while *TsWRKY33* and *TsWRKY62* differ by an amino acid.

**Figure 2:**
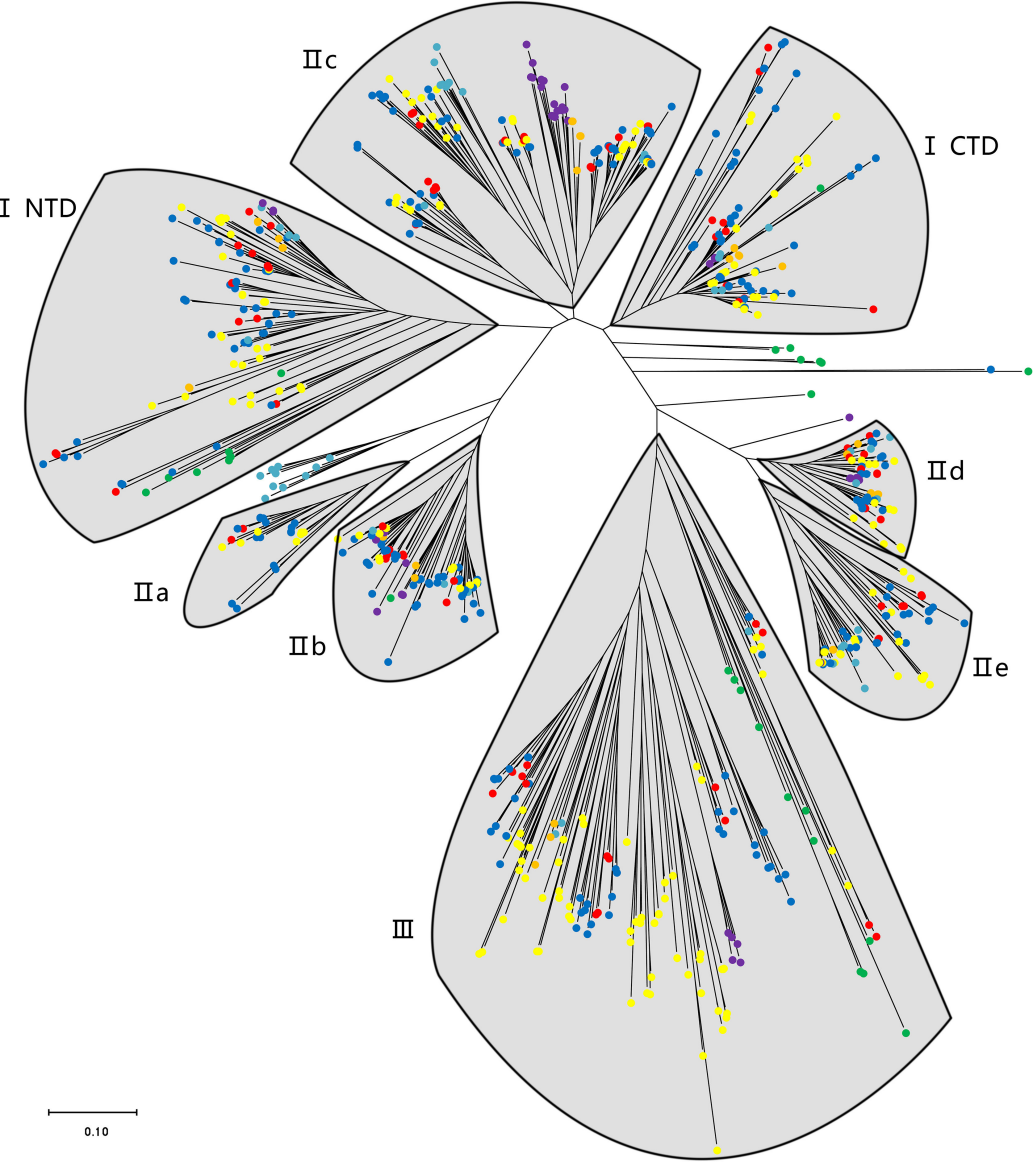
A phylogenetic tree of WRKY domains from *T. sinensis* and other species in the green lineage. The phylogenetic tree was derived from a MUSCLE alignment of WRKY domains from the following species: *Micromonas pusilla*, *Ostreococcus tauri*, *Ostreococcus lucimarinus*, *Dunaliella salina*, *Chlamydomonas reinhardtii*, *Gonium pectorale*, *Volvox carteri*, *Physcomitrella patens*, *Selaginella moellendorffii*, *Brachypodium distachyon*, *Oryza sativa*, *Glycine max*, *Arabidopsis thaliana*, and *T. sinensis*. The green, purple, orange, indigo, yellow, blue, and red dots represent unicellular green algae, bryopsida, pteridophyta, gymnosperms, monocotyledons, dicotyledons, and T. sinensis, respectively.

The phylogenetic analysis using the WRKY genes of Arabidopsis as a reference revealed that these TsWRKY genes are more precisely classified into groups I, II (a–e), and III (Eulgem et al., 2000; Zhang and Wang, 2005). Among the 78 members of the *TsWRKY* family, group II contains the most TsWRKY proteins (54), followed by group I (11) and group III (11). Additionally, each category may be subdivided into numerous subcategories. There are 11 TsWRKY proteins in group I that contain two WRKY conserved domains that are classified as N-terminal *WRKYs* (IN) or C-terminal *WRKYs* (IC) depending on their locations on the protein. Group II of the TsWRKY proteins can be divided into five subgroups, including two subgroups IIa, six subgroups IIb, 27 subgroups IIc, 10 subgroups IId, and nine subgroups IIe (**Figure S1**, **Table S2**). The 11 TsWRKY proteins in group III contain a zinc finger motif of the form C-X7-C-X23-H-X-C, which is identical to the *AtWRKY30* in Arabidopsis subgroup III (**Figure 1**). We created a broader WRKY domain dataset for phylogenetic analysis to further understand the evolution of *TsWRKYs* familly. **Figure 2** shows the neighbor-joining phylogenetic tree constructed by WRKY domains for 14 species. A scattered distribution of TsWRKYs was observed in groups I, II (a–e), and III. The TsWRKYs protein sequence branched away from algae, bryopsida, and pteridophyta but showed clustering with dicotyledons and monocotyledons within each subgroup. Interestingly, the TsWRKYs protein sequence branches tend to show close proximity between the two. For example, TsWRKY35-TsWRKY57 and TsWRKY25-TsWRKY51 pairs in group IIb, and TsWRKY13-TsWRKY15, TsWRKY21-TsWRKY67 and TsWRKY48-TsWRKY65 pairs in group IId (**Figure 2**; **Figure S2**). Group III WRKY family members could be significantly subdivided into eight clades, but all TsWRKYs proteins were found on Clade 1, 4, 6, and 8.

### 3.3 Gene structure and motif composition of *TsWRKYs* genes family

**Figure 3:**
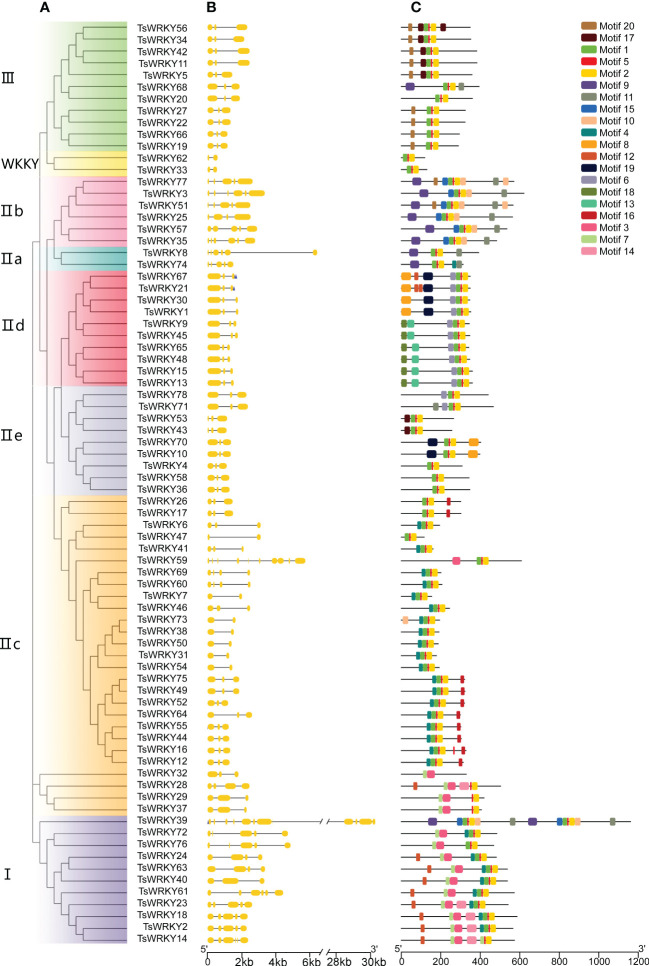
Phylogenetic tree, exon-intron distribution, and conserved protein motifs of TsWRKYs. **(A)** The TsWRKYs are divided into several groups, each with a distinct color. **(B)** Yellow rectangles and black lines represent exons and introns, respectively. **(C)** Each motif with conserved amino acid residues is represented in different color (motif 1 – 20).

**Figure 3B** depicts the particular condition of the *T. sinensis WRKY* gene structures. The number of introns, with the exception of *TsWRKY39* and *TsWRKY59*, ranges from 2 to 6, with an average of 3.59. The *TsWRKY* genes structure are composed of three exons and two introns in more than 60% (47 of the 78) of them. *TsWRKY39*, in particular, has the most exons and introns of all *TsWRKYs*, with 12 exons and 11 introns. Gene structures of genes in the same group, like IId and IIe, tend to be consistent in general.

The domain prediction results (**Figure 3C**) were validated by utilizing the MEME web server. By sequencing the TsWRKY proteins, 20 distinct motifs were discovered, comprising 8-50 amino acids. The majority of *TsWRKY*s contained motifs 1, 2, and 5, which corresponded to the DBD domain, while others contained motifs unique to each class. For instance, motif 8 is unique to group IId, while motif 10 is found only in groups IIa and IIb. Most importantly, each class had a distinct motif organization, and two genes that were tightly clustered on the tree usually exhibited identical motif patterns. Despite their heterogeneity in size and sequence, the projected WRKY domains and other conserved domains were cross-confirmed by the two combined approaches, implying that the group classifications are reliable.

### 3.4 Cis-regulatory element prediction of *TsWRKYs* genes

**Figure 4:**
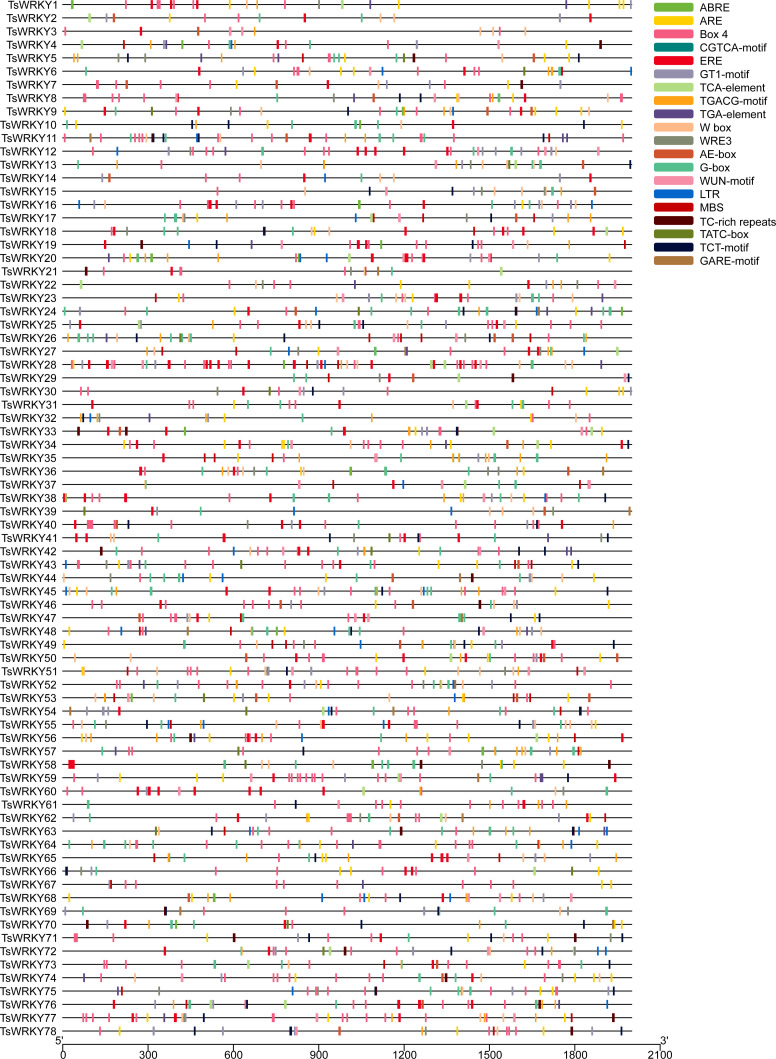
The prediction of CREs in the 2000 bp promoter upstream of the *TsWRKY genes*. The main CREs are showed in the upper right corner.

CREs are genomic sequence motifs located in the 5’ upstream region of genes that bind to motif-specific proteins and function as regulatory switches for downstream genes (Korkuć et al., 2013). As shown in **Figure 4**, the upstream 2000 bp regulatory regions of all *TsWRKYs* were extracted, several CREs were predicted using PlantCARE, and the 20 most common were visualized using TBtools software.

Our analysis revealed that *T. sinensis* contained many promoters’ core regulatory elements (CAAT-box, TATA-box), light responsive elements (Box 4, G-box, GT1-motif, AE-box, and TCT-motif), and W box elements. We observed a lot of abiotic stress responsive elements as well, such as wound-responsive elements (WUN-motif), drought-inducibility elements (MBS), dehydration, low-temp, salt stress responsive elements (DRE), low-temperature responsive elements (LTR and WRE3), and defense and stress responsive elements (TC-rich repeats). These are the hormone responsive elements: abscisic acid responsive elements (ABREs), methyl jasmonate (MeJA) responsive elements (CGTCA-motif and TGACG-motif), ethylene-responsive elements (EREs), auxin-responsive element (TGA-element), gibberellin-responsive elements (GARE-motif, P-box and TATC-box), and salicylic acid responsive element (TCA-element). Other CREs were also predicted, such as anaerobic responsive elements (AREs) and circadian control elements (circadian).

All *TsWRKYs* had at least one stress response-related CREs in this investigation. A total of 65 *TsWRKY* genes (83.3%) had one or more ABREs, which could be a sign that they have an ABA response when they are stressed. Additionally, more than 70% of *TsWRKY* genes have the EREs and AREs that have been speculated as having important promoter roles (Olive et al., 1991; Ohme-Takagi et al., 2000). *TsWRKY*27 and *TsWRKY*72 contained 16 out of 20 promoters in their promoter regions that surpass other *TsWRKY* genes. We also focused on CREs involved in wound, hypothermia, and drought responses, such as WUN-motif, LTR, MBS, and TC-rich repeats. The WRKY protein can be used efficiently in conjunction with W-box regions to activate or inhibit downstream target gene transcription (Jiang et al., 2017). It can form protein complexes with other active components, which improves transcription binding activity (Chi et al., 2013). Moreover, 62 *TsWRKY*s possessed one or even more W-boxes, implying that these *WRKY* genes are regulated by autoregulation or crossregulation (Rushton et al., 2010).

### 3.5 Chromosomal distribution and synteny analysis of *TsWRKYs* genes

**Figure 5:**
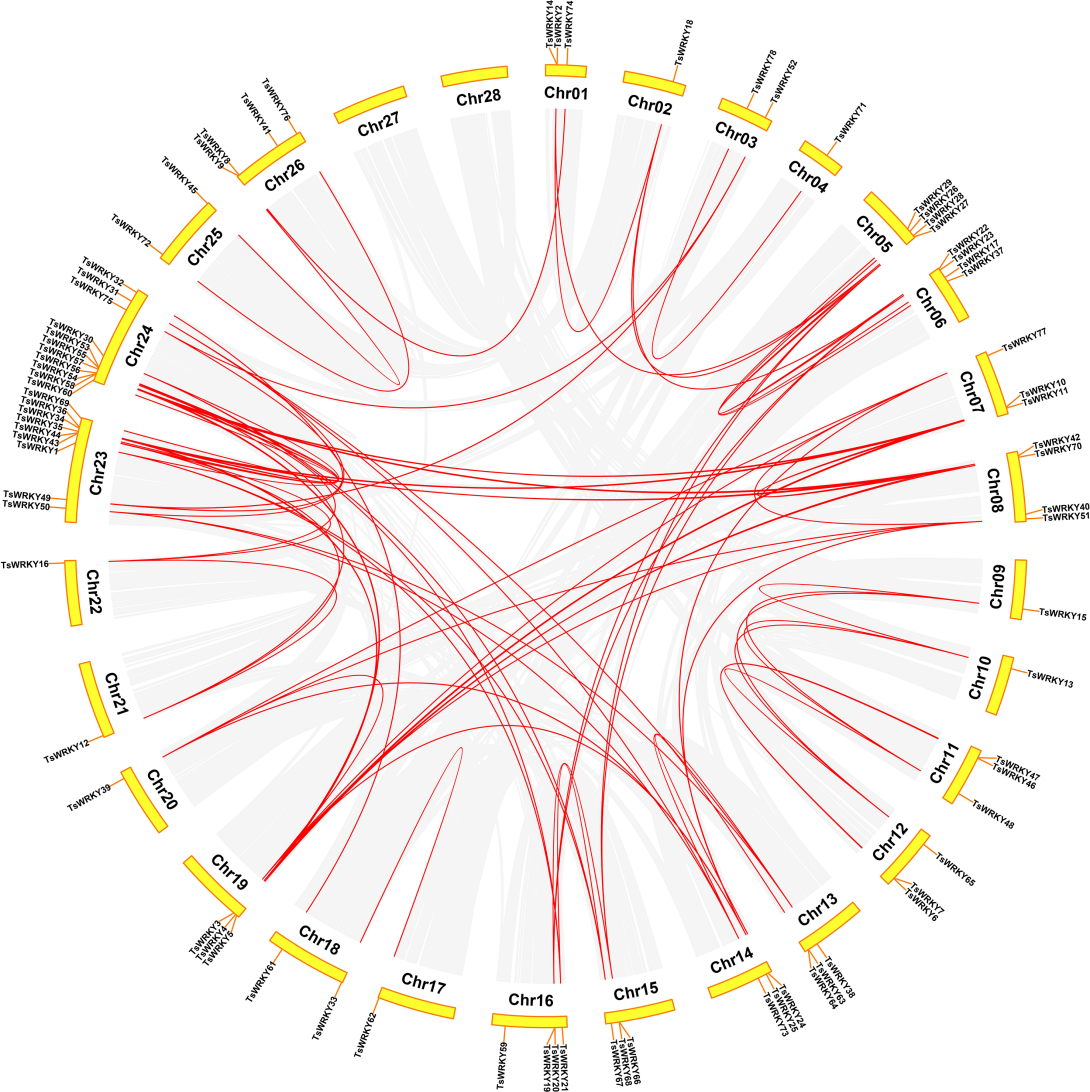
The *WRKY* genes segmental duplication in *T. sinensis*. The gray lines inside represent synteny blocks, whereas the red lines imply duplicated *WRKY* gene pairs that have been discovered.

The 78 *TsWRKY* genes were dispersed randomly throughout the 28 *T. sinensis* chromosomes (**Figure 5**). The bulk of the *TsWRKYs* were found at or at the ends of chromosomes. Of all *TsWRKYs*, 11 were identified on Chr24, scattered in several clusters, which is the largest number. On the contrary, there is only a single *TsWRKY* gene on Chr2, Chr4, Chr9, Chr10, Chr17, Chr20, Chr21, and Chr22. Tandem and segmental duplications both contribute to the generation of gene families throughout evolution (Cannon et al., 2004). Hence, we investigated the occurrences of *TsWRKY* genes duplication. On cross-referencing with Holub’s published research study, 20 *TsWRKY* genes (25.7%) were found to be tandem duplicated. Tandem duplication event is a chromosomal region within 200 kb, including multiple (two or more) members of a gene family (Holub, 2001). There were 10 distinct pairs of tandemly duplicated genes on Chr 1, 6, 11, 12, 13, 15, 16, 23, and 24. In addition to the tandem duplication events, 83 segmental duplication events involving 72 *TsWRKY* genes were discovered using the BLASTP and MCScanX approaches.

**Figure 6:**
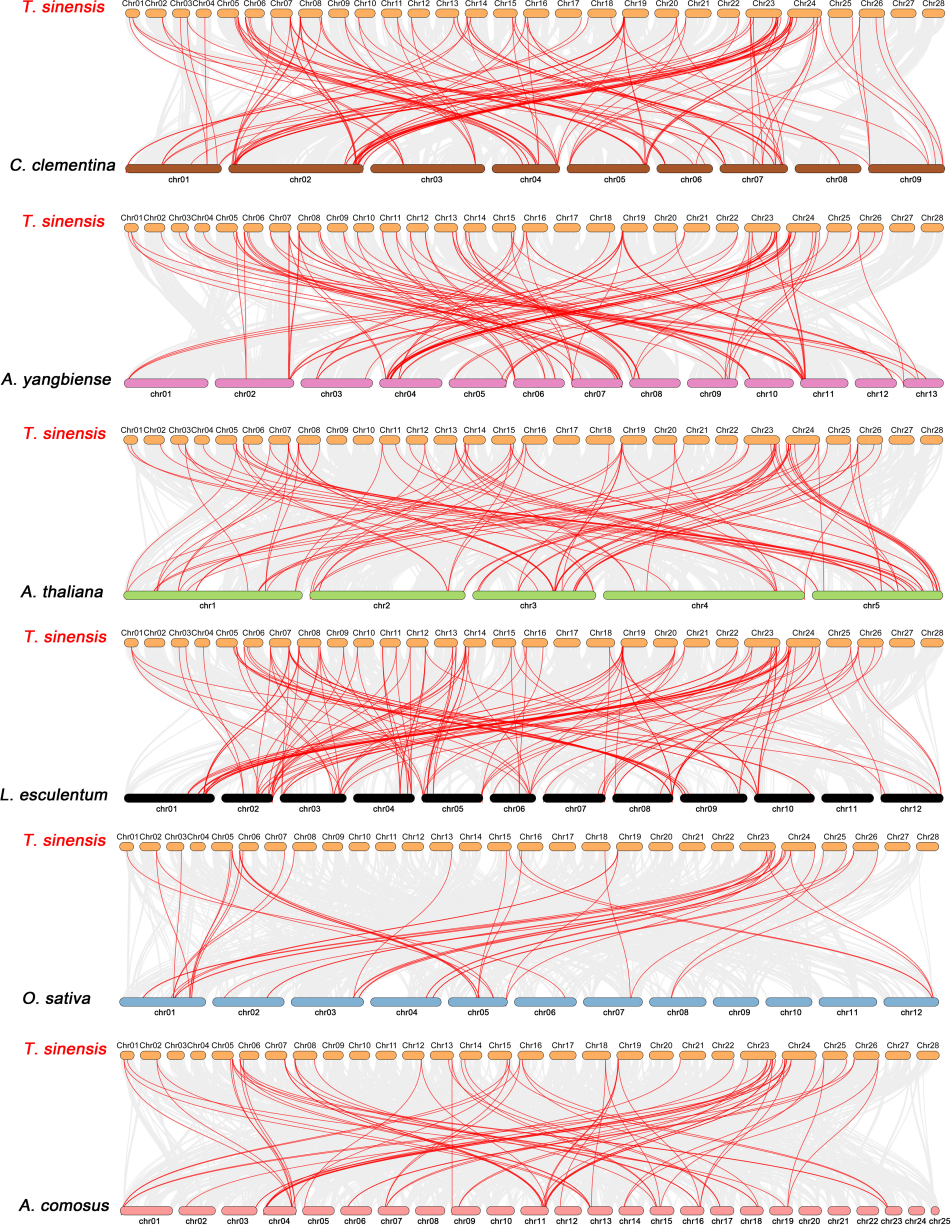
Synteny analysis of WRKY genes between *T. sinensis* and six representative plant species: *Citrus clementina* (*C. clementina*), *Acer yangbiense* (*A. yangbiense*), *Arabidopsis thaliana* (*A. thaliana*), *Lycopersicon esculentum* (*L. esculentum*), *Oryza sativa* (*O. sativa*), and *Ananas comosus* (*A. comosus*). Every horizontal bar indicates a different chromosome. The red curves represent the syntenic WRKY gene pairs, whereas the gray lines indicate the collinear blocks within *T. sinensis* and other plant genomes.

**Figure 7:**
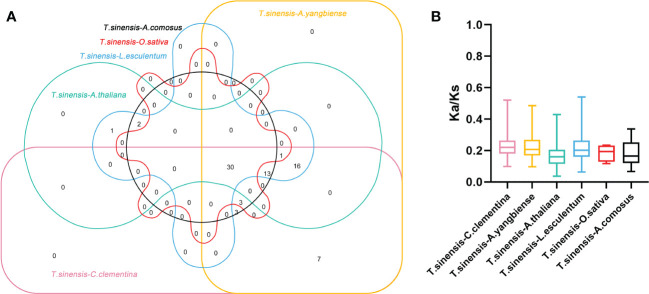
Non-redundant syntenic and evolution analysis of the *WRKY* gene families in a diversity of species. **(A)** The Venn diagram depicts syntenic *WRKY* genes found in multiple species. **(B)** The ratio of nonsynonymous to synonymous substitutions (Ka/Ks) of *WRKY* genes in *T. sinensis* and other six species: *Citrus clementina (C. clementina)*, *Acer yangbiense (A. yangbiense)*, *Arabidopsis thaliana (A. thaliana)*, *Lycopersicon esculentum (L. esculentum)*, *Oryza sativa (O. sativa)*, and *Ananas comosus (A. comosus)*.

Constructing syntenic graphs between *T. sinensis* and several typical species allows us to investigate the evolutionary clues for the *T. sinensis WRKY* gene family (**Figure 6**). The representative species consist of four dicots, *Citrus clementina*, *Acer yangbiense*, *A. thaliana*, and *Lycopersicon esculentum*, and two monocots, *O. sativa* and *Ananas comosus*. Syntenic links were found between 77 *TsWRKY* gene members and those in citrus (73), maple (70), tomato (70), Arabidopsis (63), pineapple (51), and rice (34). There were 136, 129, 113, 101, 73, and 45 orthologous pairings between the six species (tomato, citrus, maple, Arabidopsis, pineapple, and rice), respectively. In general, the *TsWRKYs* comprised more syntenic gene pairs in dicots than in monocots. *C. clementina* and *A. yangbiense*, well-known members of Sapindales, show greater synteny with *T. sinensis*, which belonged to Sapindales*. *Notably, in the interactive Venn map of *WRKY* genes across species (**Figure 7A**), 30 *TsWRKY* genes shared syntenic WRKY gene pairings with all six species, implying that these orthologous pairs existed prior to the ancestral split. Certain *TsWRKY* genes were shown to relate to 3, 4, or 5 collinear gene pairs (between *T. sinensis* and maple/citrus/tomato *WRKY* genes), indicating the possibility that these *TsWRKY* genes have significant roles in the evolution of the *WRKY* gene family. Syntenic gene pairings between *T. sinensis* and other species may be important for elucidating *WRKY* gene evolution. The Ka/Ks (non-synonymous substitution/synonymous substitution) ratios of the *WRKY* orthologous gene pairs of six species were computed to assess the evolutionary constraints operating on the *T. sinensis WRKY* gene family. **Figure 7B** shows that almost all *TsWRKY* orthologous gene pairs had Ka/Ks < 1. As a result, we speculated that the *T. sinensis WRKY* gene family may have been subjected to significant purifying selection forces throughout evolution (Hurst, 2002).

### 3.6 Expression patterns of *TsWRKYs* genes and terpenoid synthases genes

**Figure 8:**
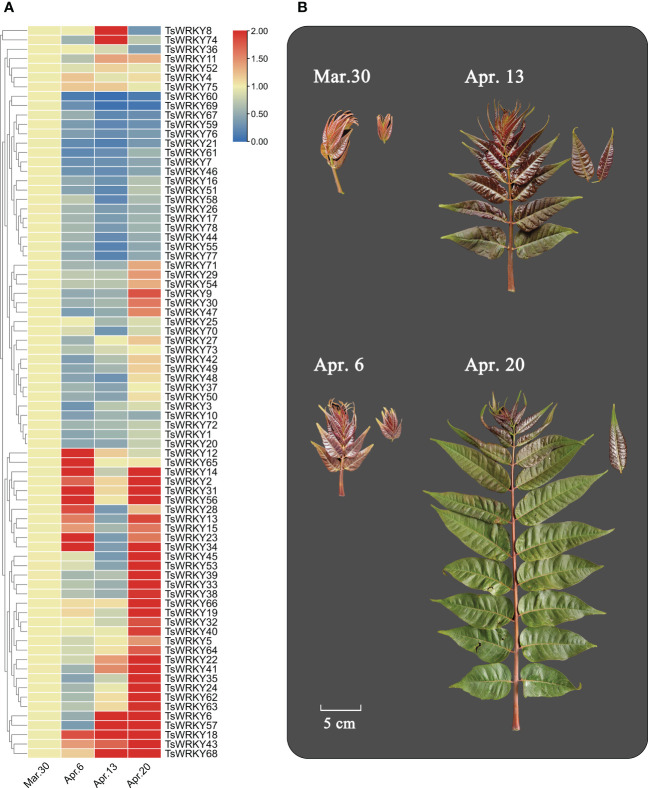
Differential transcription of *TsWRKY* genes in four different sampling periods (March 30, April 6, April 13, and April 20, 2021). **(A)** Blue and red indicate lower and higher transcript abundance, respectively, compared to initial data (March 30). **(B)** Samples of *T. sinensis* leaves from four periods.

Six main expression patterns of *TsWRKY* genes were observed (**Figure 8**). *TsWRKY*8 and 74 were upregulated at first and then downregulated, while *TsWRKY12* and *TsWRKY65* showed the opposite trend. Suppressed expression patterns were seen in 18 *TsWRKY* genes (7, 16, 17, 21, 26, 44, 46, 51, 55, 58, 59, 60, 61, 67, 69, 76, 77, and 78), while upregulated expressions were observed in 22 *TsWRKY* genes (5, 6, 18, 19, 22, 24, 32, 33, 35, 38, 39, 40, 41, 43, 45, 53, 57, 62, 63, 64, 66, and 68). Furthermore, the expression of nine *TsWRKY* genes (2, 13, 14, 15, 23, 28, 31, 34, and 56) were first upregulated, then downregulated, and then upregulated to a high level. The other *TsWRKY* genes (1, 3, 4, 9, 10, 11, 20, 25, 27, 29, 30, 36, 37, 42, 47, 48, 49, 50, 52, 54, 70, 71, 72, 73, 75, and 78) were not significantly altered (fold change ≥ 2) at different stages.

**Figure 9:**
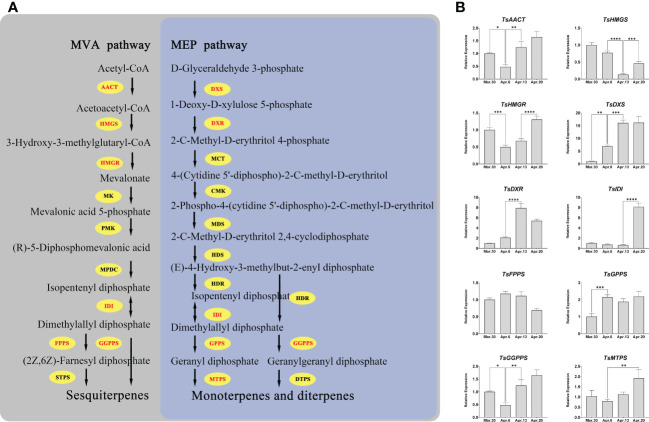
Pathways of terpenoid synthesis and the expression patterns of several key genes. **(A)** The main terpenoid biosynthesis pathway in plants; *AACT*, acetoacetyl-CoA thiolase; *HMGS*, 3-hydroxy-3-methylglutaryl-CoA synthase; *HMGR*, 3-hydroxy-3-methylglutaryl-CoA reductase; *IDI*, isopentenyl diphosphate isomerase; *FPPS*, farnesyl diphosphate synthase; *DXS*, 1-deoxyd-xylulose 5-phosphate synthase; *DXR*, 1-deoxy-D-xylulose 5-phosphate reductoisomerase; *GPPS*, geranyl diphosphate synthase; *GGPPS*, geranylgeranyl diphosphate synthases; *PMK*, phosphomevalonate kinase; *MK*, mevalonate kinase; *MPDC*, mevalonate diphosphate decarboxylase; *MCT*, 2-C-methyl-D-erythritol 4-phosphate cytidylyltransferase; *CMK*, 4-(cytidine 5’-diphospho)-2-C-methyl-D-erythritol kinase; *MDS*, 2-C-methyld-erythritol 2,4-cyclodiphosphate synthase; *HDS*, **(E)**-4-hydroxy-3-methylbut-2-enyl diphosphate synthase; *HDR*, **(E)**-4-hydroxy-3-methylbut-2-enyl diphosphate reductase; *STPS*, sesquiterpene synthase; *MTPS*, monoterpene synthase; *DTPS*, diterpene synthase. **(B)** *, **, ***, and **** mean a significant difference at P < 0.05, P < 0.01, P < 0.001, and P < 0.0001, respectively.

We investigated the expression patterns of the genes involved in terpenoid biosynthesis in order to unravel the regulatory mechanism of terpenoid accumulation patterns in various developmental stages of *T. sinensis*. All of the key genes, except for *TsFPPS*, showed significant changes (fold change ≥ 2) in expression during development (**Figure 9B**). After April 6, the expressions of *TsAACT*, *TsHMGS*, *TsHMGR*, *TsDXS*, and *TsDXR* all changed distinctly. Notably, *TsIDI*, *TsDXS*, and *TsDXR* are more than five-fold changes in expression over four periods.

### 3.7 The co-expression network of *TsWRKYs* genes and terpenoid synthesis genes

**Figure 10:**
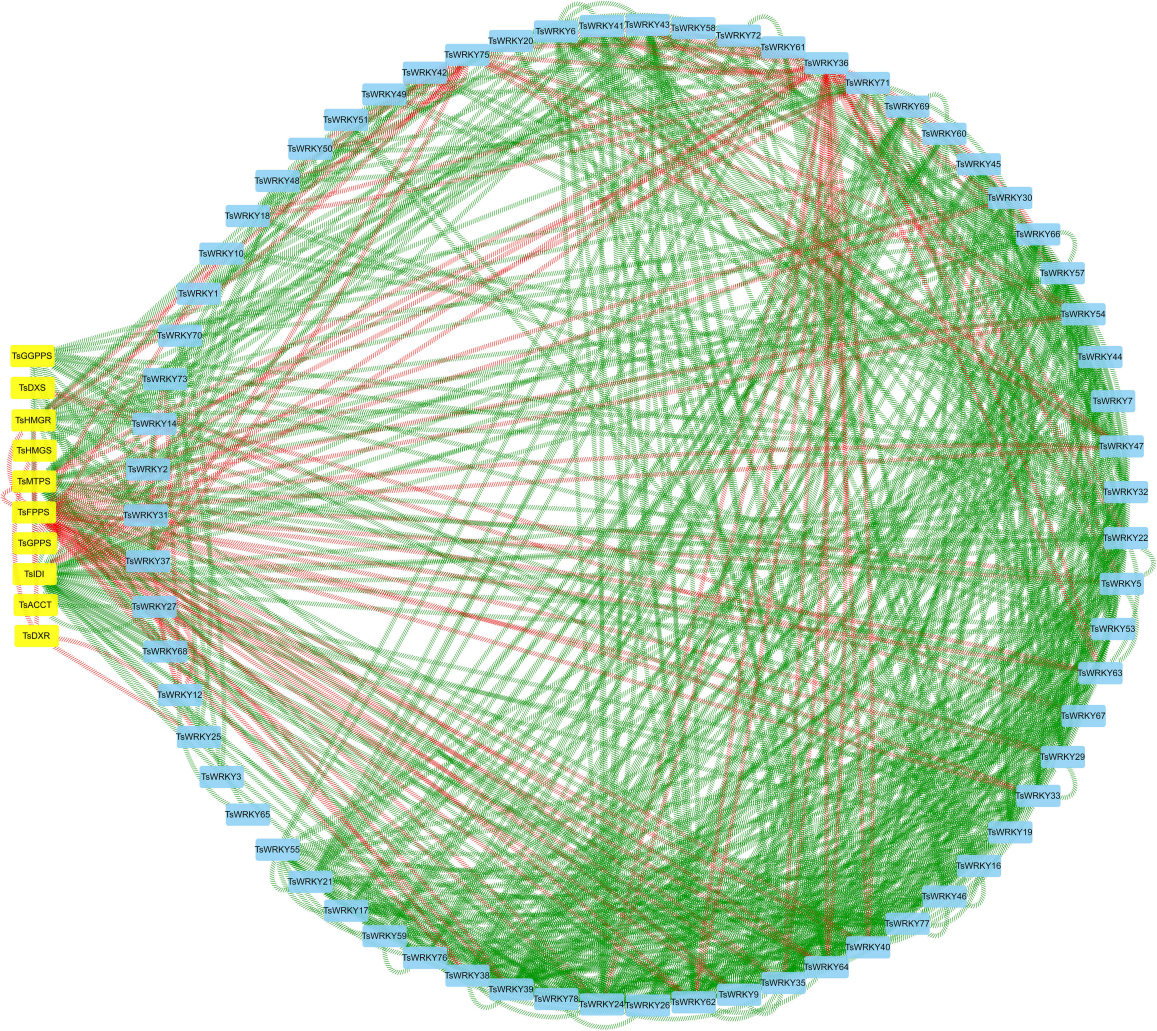
Correlation analysis of *TsWRKYs* and terpenoid synthesis genes (Pearson correlation coefficient > 0.95). The square nodes colored in blue are *TsWRKYs*, the square nodes colored in yellow are terpenoid synthesis genes, the green line represents positive correlation, and the red line represents negative correlation.

The *WRKYs* usually control the expression of terpenoid synthesis genes by activating or repressing their promoters, thereby regulating the accumulation of terpenoids. We constructed a co-expression network of *TsWRKYs* with terpenoid synthesis genes (**Table S3**; **Figure 10**). The results showed that *TsFPPS*, *TsIDI*, *TsMTPS*, *TsWRKY9*, *TsWRKY24*, *TsWRKY35*, *TsWRKY38*, *TsWRKY39*, *TsWRKY62*, and *TsWRKY64* are possible core members of the terpenoid synthesis co-expression network. *TsFPPS*, *TsWRKY36*, and *TsWRKY75* were negatively correlated with other genes. By contrast, *TsWRKY24*, *TsWRKY35*, *TsWRKY39*, and *TsWRKY40* are almost all positive correlated factors.

## 4 Discussion

Since the first WRKY proteins were identified, *WRKY* TFs have been well recognized in plants for their regulating functions in defense against abiotic and biotic stresses, growth and development, and secondary metabolism (Schluttenhofer and Yuan, 2015; Jiang et al., 2017; Abeysinghe et al., 2019). Terpenes are the signature volatile components of *T. sinensis*, and *WRKYs* are significantly involved in regulating the terpene pathway (Yang et al., 2012; Zhao et al., 2022). Neither the identification of the *WRKY* gene nor the regulation of terpenoids, the most iconic volatile substances in T. sinensis, has been reported. We describe *WRKY* TFs in *T. sinensi*s and present the associated regulatory network of terpene biosynthesis.

### 4.1 Evolutionary characteristics of *WRKY* TFs in *T. sinensis*

Variation in the number of gene family members is a key mechanism for shaping adaptive natural variation during the evolution of species (Guo, 2013). We discovered 78 proper *TsWRKY* genes in this investigation. The results of the neighbour-joining phylogenetic tree of 14 species constructed with the WRKY protein sequence’s conserved domain indicate that they can be divided into three major groups (I–III) and five subgroups (IIa–IIe). *WRKY* TFs diverge early in the green lineage and *TsWRKYs* are more closely related to dicotyledons and monocotyledons. The number of presumed *TsWRKYs* are comparable to the count of *WRKY* genes in *Fagopyrum tataricum* (78) (He et al., 2019), and it is somewhat lower than those in *S. lycopersicum* L. (81) (Huang et al., 2012), but much lower than those in *O. sativa L. ssp. indica* (102) (Ross et al., 2007) and *Glycine max* (174) (Yang et al., 2017). These findings corroborated previous research that suggested herbaceous plants tend to have a larger number of WRKY genes than woody plants (Wu et al., 2016).

The variety of gene structures reflects the historical evidence of gene family evolution and serves as the foundation for phylogenetic categorization (Xiao et al., 2017). Wheat and tea plants have 0–5 introns and 0–11 introns, respectively, whereas *TsWRKYs* have 2–12 introns, suggesting that *TsWRKYs* have abundant gene structural variation (Ning et al., 2017; Wang et al., 2019). The intron-exon distribution pattern is comparable across members of the same subfamily, which is the basis for functional similarity among members of the same evolutionary group (Li et al., 2020a). For example, the number of introns in almost all members of group III is 2. Furthermore, *TsWRKYs* from group I contain many more introns than other groups, which implies that it is more likely that other groups came from group I (Chen et al., 2017a). Twenty conserved motifs were discovered in 78 TsWRKY proteins (**Figure 3**), with motifs 1 and 2 belonging to the WRKY conserved domains. Partiular motifs that only arose in one group, such as motif 10 in group IIa and group IIb proteins and motif 8 in subgroup IId, which has yet to be characterized for some roles, should be given additional attention.

According to a recent study, dicotyledons have experienced less evolutionary loss of the WRKY conserved domain than monocotyledons (Wei et al., 2012). This occurrence was validated in this investigation, with the majority of *TsWRKYs* having the conserved heptapeptid WRKYGQK motif, despite two *TsWRKY* genes having the variants WKKYGQK (TsWRKY33 and TsWRKY62, Group WKKY) (**Figure 1**). The WRKY proteins demonstrate a propensity for binding to W-box elements, and WRKYGQK motif changes may affect DNA-binding interactions with downstream genes, as previously discovered. As a result, additional exploration of the functional and binding properties of these two WRKY proteins is required (Chen et al., 2017a).

How did the number of members of the *WRKY* family expand from 1 in the unicellular green algae to 78 in the *T. sinensis*? The analysis of segmental and tandem duplications contributed to revealing the number and function of the *TsWRKY* gene family. Based on the chromosome distribution and synlinearity analysis results of the *TsWRKYs*, 83 segmental duplication events within 72 *TsWRKY* genes were observed, while tandem duplication events existed. We found that 20 out of the 78 genes (25.6%) in this family are tandem repeats in *T. sinensis*, suggesting that the abundance of tandem repeats may be a possible reason for the larger number of *TsWRKYs*. In addition, whole-genome duplication (WGD) events often lead to the growth of gene families, which is common in the evolution of angiosperms. Previous study revealed that *T. sinensis* has a large number of gene duplications and that WGD events happened approximately 7.8 and 71.5 million years ago (Mya) (Ji et al., 2021). We determined that, whereas some *TsWRKY* genes are the result of tandem duplication, segment duplication events are the driving force behind gene family evolution. The three basic evolutionary mechanisms are segmental duplication, tandem duplication, and transposition events like retroposition and replicative transposition. Individual gene duplication, chromosomal segment duplication, and even complete genome duplication supply the fresh materials required for gene generation (Yu et al., 2005). Gene duplication, which is associated with the generation of new gene functions, is one of the primary driving forces underlying genome evolution and also is essential to plant adaptive evolution (Moore and Purugganan, 2003; Kong et al., 2007). Tandem duplications produce highly diverse duplicates that have lineage-specific functions (Ezoe et al., 2020), and new research tea plants suggests that tandem duplication of genes plays an active part in flavor accumulation (Wang et al., 2021). Additionally, we speculated that the *TsWRKY* gene family may have been subjected to significant purifying selection forces throughout evolution because almost all *TsWRKYs* orthologous gene pairs had Ka/Ks<1. Divergence is a key feature of the evolution of paralogous homologous genes and DNA segments that make up fixed repeats. However, selection on copies of paralogous homologs would be relaxed in the case of full redundancy, which is when any number of functional copies of a gene give the same fitness. Therefore, this negative selection of *TsWRKYs* are associated with the post-fixation evolution of gene duplications.

### 4.2 Regulation of terpene biosynthesis by *TsWRKYs* genes in *T. sinensis*

The main volatile aromatic compounds of *T. sinensis* are terpenes (isopentene, monoterpenes, and sesquiterpenes), phenyl/phenylpropanes, and fatty acid derivatives. In *T. sinensis*, the terpenes have been demonstrated as the most important volatile compounds. The aroma of *T. sinensis* leaves is an important factor in determining its quality and an important criterion for measuring its economic value. During the germination and maturation process of *T. sinensis* tender buds, aromatic substances are gradually synthesized and accumulated. The results of real-time quantitative PCR revealed that 67.9 percent of *TsWRKY* had significantly varied transcript levels during the four stages of budding. Although little is known about the transcriptional regulatory network controlling terpenes synthesis, most of the identified regulators are *WRKY* TFs (Patra et al., 2013). It is reasonable to conclude that *TsWRKYs* expression patterns and the buildup of aromatic compounds are related. Therefore, we further analyzed the expression patterns of terpenoid synthase genes and detected that multiple genes of the MVA and MEP pathways are involved in regulating terpenoid accumulation. *TsWRKYs* showed a high correlation with the expression trends of 10 terpene synthesis genes. For example, *TsFPPS*, *TsIDI*, *TsMTPS*, *TsWRKY9*, *TsWRKY24*, and *TsWRKY35* may coordinately regulate terpene synthesis.

*WRKY* genes are essential regulators of secondary metabolite production in plants (Luo et al., 2022), while their regulatory functions varied substantially due to distinct binding mechanisms (Li et al., 2020b). Evidence suggests that certain *WRKYs*, alone or in concert with other transcription factors, govern the biosynthesis of valuable natural products (Hsin et al., 2022). There have been several studies on the regulatory effects of *WRKYs* upon the activation or repression of genes involved in plant terpenoid production. Individual *WRKY* can be associated with a number of regulatory mechanisms, as *SlWRKY73* transactivates the *SlTPS3*, *SlTPS5*, and *SlTPS7* monoterpene synthase genes in tomato (*S. lycopersicum*) (Spyropoulou et al., 2014). Gossypol (sesquiterpene phytoalexins) in *Gossypium arboretum* (Xu et al., 2004), DP (diterpenoid phytoalexin) in rice (Akagi et al., 2014), Artemisinin (a type of sesquiterpene lactone) in *Artemisia annua* (Chen et al., 2017b), ginsenosides (a group of triterpene) in *Panax quinquefolius* (Sun et al., 2013), and tanshinone (one category of bioactive diterpenes) in *Salvia miltiorrhiza* are all regulated by *WRKYs* (Cao et al., 2018). From correlative analysis in sweet *Osmanthus fragrans*, it is speculated that the *OfWRKY* gene participates in aroma synthesis by regulating the synthesis of monoterpene volatiles, and that the expression of *OfWRKYs* are closely related to monoterpene synthesis (Ding et al., 2019). Heterologous expression of *WRKY* and *MYC2* in *Salvia sclarea* causes coactivation of MEP-biosynthetic genes and accumulation of abietane diterpenes (Alfieri et al., 2018). The co-expression network between several key genes for terpene biosynthesis and *TsWRKYs* provides important insights into the terpene biosynthesis pathway in *T. sinensis*. It helps to further characterize the functions of candidate *WRKY* gene families in *T. sinensis* and provide new ideas for agronomic genetic improvement and quality variety breeding.

## 5 Conclusions

The 78 proper *TsWRKYs* were discovered in this investigation. Segment duplication events are determined to be the driving force behind the expansion of the *TsWRKYs* gene family. TsWRKYs proteins may have been subjected to significant purifying selection forces throughout evolution. Several *TsWRKYs* that may be involved in regulating terpenoid accumulation in the MVA and MEP pathways were identified. In summary, our study provides comprehensive information on *TsWRKYs* and could facilitate further research into the functions of *TsWRKYs* in regulating the synthesis of volatile aromatic compounds and improving the aroma of edible leaves based on an understanding of the regulatory network.

## Data availability statement

The datasets presented in this study can be found in online repositories. The names of the repository/repositories and accession number(s) can be found in the article/**Supplementary Material**.

## Author contributions

LR, TG and XC conceived and designed the experiments. WW and DY performed the experiments. LR and TG analyzed the data. XD and ZM contributed reagents/materials/analysis tools. LR and WW wrote the paper. All authors contributed to the article and approved the submitted version.

## Footnotes

1
http://www.arabidopsis.org/

2
https://db.cngb.org/search/project/CNP0000958/

3
http://pfam.xfam.org/

4
http://smart.embl-heidelberg.de/

5
https://www.ncbi.nlm.nih.gov/cdd/

6
http://web.expasy.org/protparam/

7
http://www.softberry.com/

8
http://gsds.gao-lab.org/

9
https://meme-suite.org/meme/tools/meme/

10
http://bioinformatics.psb.ugent.be/webtools/plantcare/html/

11
https://www.ncbi.nlm.nih.gov/

## Supplementary material

Supplementary Figure 1 A phylogenetic tree of WRKY domains from *T. sinensis* and *A. thaliana.*

Supplementary Figure 2 A phylogenetic tree of WRKY domains from *T. sinensis* and other species in the green lineage.

## References

[B1] Abeysinghe J. K. Lam K. M. Ng D. W. K. (2019). Differential regulation and interaction of homoeologous *WRKY 18* and *WRKY 40* in *Arabidopsis* allotetraploids and biotic stress responses. Plant J. 97 (2), 352–367. doi: 10.1111/tpj.14124 30307072

[B2] Agarwal P. Reddy M. Chikara J. (2011). *WRKY*: its structure, evolutionary relationship, DNA-binding selectivity, role in stress tolerance and development of plants. Mol. Biol. Rep. 38 (6), 3883–3896. doi: 10.1007/s11033-010-0504-5 21107718

[B3] Akagi A. Fukushima S. Okada K. Jiang C.-J. Yoshida R. Nakayama A.. (2014). *WRKY45*-dependent priming of diterpenoid phytoalexin biosynthesis in rice and the role of cytokinin in triggering the reaction. Plant Mol. Biol. 86 (1), 171–183. doi: 10.1007/s11103-014-0221-x 25033935 PMC4133022

[B4] Alfieri M. Vaccaro M. C. Cappetta E. Ambrosone A. De Tommasi N. Leone A. (2018). Coactivation of MEP-biosynthetic genes and accumulation of abietane diterpenes in *Salvia sclarea* by heterologous expression of *WRKY* and *MYC2* transcription factors. Sci. Rep. 8 (1), 11009. doi: 10.1038/s41598-018-29389-4 30030474 PMC6054658

[B5] Bailey T. L. Johnson J. Grant C. E. Noble W. S. (2015). The MEME suite. Nucleic Acids Res. 43 (W1), W39–W49. doi: 10.1093/nar/gkv416 25953851 PMC4489269

[B6] Cannon S. B. Mitra A. Baumgarten A. Young N. D. May G. (2004). The roles of segmental and tandem gene duplication in the evolution of large gene families in *Arabidopsis thaliana* . BMC Plant Biol. 4 (1), 1–21. doi: 10.1186/1471-2229-4-10 15171794 PMC446195

[B7] Cao W. Wang Y. Shi M. Hao X. Zhao W. Wang Y.. (2018). Transcription factor *SmWRKY1* positively promotes the biosynthesis of tanshinones in *Salvia miltiorrhiza* . Front. Plant Sci. 9. doi: 10.3389/fpls.2018.00554 PMC5934499 29755494

[B8] Chen C. Chen H. Zhang Y. Thomas H. R. Frank M. H. He Y.. (2020). TBtools: an integrative toolkit developed for interactive analyses of big biological data. Mol. Plant 13 (8), 1194–1202. doi: 10.1016/j.molp.2020.06.009 32585190

[B9] Chen J. Gao T. Wan S. Zhang Y. Yang J. Yu Y.. (2018). Genome-wide identification, classification and expression analysis of the *HSP* gene superfamily in tea plant (*Camellia sinensis*). Int. J. Mol. Sci. 19 (9), 2633. doi: 10.3390/ijms19092633 30189657 PMC6164807

[B10] Cheng Y. Jalal A. G. Yu J. Yao Z. Ruan M. Ye Q.. (2016). Putative *WRKYs* associated with regulation of fruit ripening revealed by detailed expression analysis of the *WRKY* gene family in pepper. Sci. Rep. 6 (1), 1–11. doi: 10.1038/srep39000 27991526 PMC5171846

[B11] Chen F. Hu Y. Vannozzi A. Wu K. Cai H. Qin Y.. (2017a). The *WRKY* transcription factor family in model plants and crops. Crit. Rev. Plant Sci. 36 (5-6), 311–335. doi: 10.1080/07352689.2018.1441103

[B12] Chen H. Lai Z. Shi J. Xiao Y. Chen Z. Xu X. (2010). Roles of *Arabidopsis WRKY18*, *WRKY40* and *WRKY60* transcription factors in plant responses to abscisic acid and abiotic stress. BMC Plant Biol. 10 (1), 281. doi: 10.1186/1471-2229-10-281 21167067 PMC3023790

[B13] Chen M. Yan T. Shen Q. Lu X. Pan Q. Huang Y.. (2017b). Glandular trichome-specific *WRKY* 1 promotes artemisinin biosynthesis in *Artemisia annua* . New Phytol. 214 (1), 304–316. doi: 10.1111/nph.14373 28001315

[B14] Chi Y. Yang Y. Zhou Y. Zhou J. Fan B. Yu J. Q.. (2013). Protein–protein interactions in the regulation of *WRKY* transcription factors. Mol. Plant 6 (2), 287–300. doi: 10.1093/mp/sst026 23455420

[B15] Dang F. Wang Y. Yu L. Eulgem T. Lai Y. Liu Z.. (2013). *CaWRKY40*, a WRKY protein of pepper, plays an important role in the regulation of tolerance to heat stress and resistance to *Ralstonia solanacearum* infection. Plant Cell Environ. 36 (4), 757–774. doi: 10.1111/pce.12011 22994555

[B16] Ding W. Ouyang Q. Li Y. Shi T. Li L. Yang X.. (2019). Genome-wide investigation of *WRKY* transcription factors in sweet osmanthus and their potential regulation of aroma synthesis. Tree Physiol. 40 (4), 557–572. doi: 10.1093/treephys/tpz129 31860707

[B17] Dong X. Zhu Y. Bao G. Hu F. Qin G. (2013). New limonoids and a dihydrobenzofuran norlignan from the roots of *Toona sinensis* . Molecules 18 (3), 2840–2850. doi: 10.3390/molecules18032840 23455673 PMC6269753

[B18] Dou L. Zhang X. Pang C. Song M. Wei H. Fan S.. (2014). Genome-wide analysis of the *WRKY* gene family in cotton. Mol. Genet. Genomics 289 (6), 1103–1121. doi: 10.1007/s00438-014-0872-y 24942461

[B19] Eulgem T. Rushton P. J. Robatzek S. Somssich I. E. (2000). The *WRKY* superfamily of plant transcription factors. Trends Plant Sci. 5 (5), 199–206. doi: 10.1016/s1360-1385(00)01600-9 10785665

[B20] Ezoe A. Shirai K. Hanada K. (2020). Degree of functional divergence in duplicates is associated with distinct roles in plant evolution. Mol. Biol. Evol. 38 (4), 1447–1459. doi: 10.1093/molbev/msaa302 PMC8042753 33290522

[B21] Fan Z. Tan X. Shan W. Kuang J. Lu W. Chen J. (2017). *BrWRKY65*, a *WRKY* transcription factor, is involved in regulating three leaf senescence-associated genes in Chinese flowering cabbage. Int. J. Mol. Sci. 18 (6), 1228. doi: 10.3390/ijms18061228 28594365 PMC5486051

[B22] Gasteiger E. Hoogland C. Gattiker A. Wilkins M. R. Appel R. D. Bairoch A. (2005). Protein identification and analysis tools on the ExPASy server. Proteomics Protoc. Handb., 571–607. doi: 10.1385/1-59259-890-0:571

[B23] Goyal P. Manzoor M. M. Vishwakarma R. A. Sharma D. Dhar M. K. Gupta S. (2020). A comprehensive transcriptome-wide identification and screening of *WRKY* gene family engaged in abiotic stress in *Glycyrrhiza glabra* . Sci. Rep. 10 (1), 1–18. doi: 10.1038/s41598-019-57232-x 31941983 PMC6962277

[B24] Guo Y. L. (2013). Gene family evolution in green plants with emphasis on the origination and evolution of *Arabidopsis thaliana* genes. Plant J. 73 (6), 941–951. doi: 10.1111/tpj.12089 23216999

[B25] He X. Li J. Chen Y. Yang J. Chen X. (2019). Genome-wide analysis of the *WRKY* gene family and its response to abiotic stress in buckwheat (*Fagopyrum tataricum*). Open Life Sci. 14 (1), 80–96. doi: 10.1515/biol-2019-0010 33817140 PMC7874777

[B26] Holub E. B. (2001). The arms race is ancient history in *Arabidopsis*, the wildflower. Nat. Rev. Genet. 2 (7), 516–527. doi: 10.1038/35080508 11433358

[B27] Hsin K. Hsieh M. Lee Y. Lin K. Cheng Y. (2022). Insight into the phylogeny and binding ability of *WRKY* transcription factors. Int. J. Mol. Sci. 23 (5), 2895. doi: 10.3390/ijms23052895 35270037 PMC8911475

[B28] Huang S. Gao Y. Liu J. Peng X. Niu X. Fei Z.. (2012). Genome-wide analysis of *WRKY* transcription factors in *Solanum lycopersicum* . Mol. Genet. Genomics 287 (6), 495–513. doi: 10.1007/s00438-012-0696-6 22570076

[B29] Hu B. Jin J. Guo A. Zhang H. Luo J. Gao G. (2015). GSDS 2.0: an upgraded gene feature visualization server. Bioinformatics 31 (8), 1296–1297. doi: 10.1093/bioinformatics/btu817 25504850 PMC4393523

[B30] Hurst L. D. (2002). The Ka/Ks ratio: diagnosing the form of sequence evolution. Trends Genet. 18 (9), 486–486. doi: 10.1016/s0168-9525(02)02722-1 12175810

[B31] Ishiguro S. Nakamura K. (1994). Characterization of a cDNA encoding a novel DNA-binding protein, *SPF1*, that recognizes *SP8* sequences in the 5' upstream regions of genes coding for sporamin and β-amylase from sweet potato. Mol. Gen. Genet. 244 (6), 563–571. doi: 10.1007/BF00282746 7969025

[B32] Jiang J. Ma S. Ye N. Jiang M. Cao J. Zhang J. (2017). WRKY transcription factors in plant responses to stresses. J. Integr. Plant Biol. 59 (2), 86–101. doi: 10.1111/jipb.12513 27995748

[B33] Jia C. Wang Z. Wang J. Miao H. Zhang J. Xu B.. (2022). Genome-wide analysis of the banana *WRKY* transcription factor gene family closely related to fruit r ipening and stress. Plants 11 (5), 662. doi: 10.3390/plants11050662 35270130 PMC8912484

[B34] Ji Y. T. Xiu Z. Chen C. H. Wang Y. Yang J. X. Sui J. J.. (2021). Long read sequencing of *Toona sinensis* (A. juss) roem: A chromosome-level reference genome for the family *Meliaceae* . Mol. Ecol. Resour. 21 (4), 1243–1255. doi: 10.1111/1755-0998.13318 33421343

[B35] Kong H. Landherr L. L. Frohlich M. W. Leebens-Mack J. Ma H. DePamphilis CW (2007). Patterns of gene duplication in the plant *SKP1* gene family in angiosperms: evidence for multiple mechanisms of rapid gene birth. Plant J. 50 (5), 873–885. doi: 10.1111/j.1365-313X.2007.03097.x 17470057

[B36] Korkuć P. Schippers J. H. M. Walther D. (2013). Characterization and identification of cis-regulatory elements in *Arabidopsis* based on single-nucleotide polymorphism information. Plant Physiol. 164 (1), 181–200. doi: 10.1104/pp.113.229716 24204023 PMC3875800

[B37] Krzywinski M. Schein J. Birol I. Connors J. Gascoyne R. Horsman D.. (2009). Circos: an information aesthetic for comparative genomics. Genome Res. 19 (9), 1639–1645. doi: 10.1101/gr.092759.109 19541911 PMC2752132

[B38] Kumar S. Stecher G. Tamura K. (2016). MEGA7: molecular evolutionary genetics analysis version 7.0 for bigger datasets. Mol. Biol. Evol. 33 (7), 1870–1874. doi: 10.1093/molbev/msw054 27004904 PMC8210823

[B39] Lescot M. Déhais P. Thijs G. Marchal K. Moreau Y. Van de Peer Y.. (2002). PlantCARE, a database of plant cis-acting regulatory elements and a portal to tools for in silico analysis of promoter sequences. Nucleic Acids Res. 30 (1), 325–327. doi: 10.1093/nar/30.1.325 11752327 PMC99092

[B40] Letunic I. Khedkar S. Bork P. (2021). SMART: recent updates, new developments and status in 2020. Nucleic Acids Res. 49 (D1), D458–D460. doi: 10.1093/nar/gkaa937 33104802 PMC7778883

[B41] Li X. He L. An X. Yu K. Meng N. Duan C.. (2020b). *VviWRKY40*, a *WRKY* transcription factor, regulates glycosylated monoterpenoid production by *VviGT14* in grape berry. Genes 11 (5), 485. doi: 10.3390/genes11050485 32365554 PMC7290806

[B42] Li D. Liu P. Yu J. Wang L. Dossa K. Zhang Y.. (2017). Genome-wide analysis of *WRKY* gene family in the sesame genome and identification of the *WRKY* genes involved in responses to abiotic stresses. BMC Plant Biol. 17 (1), 1–19. doi: 10.1186/s12870-017-1099-y 28893196 PMC5594535

[B43] Li W. Wang H. Yu D. (2016). *Arabidopsis WRKY* transcription factors *WRKY12* and *WRKY13* oppositely regulate flowering under short-day conditions. Mol. Plant 9 (11), 1492–1503. doi: 10.1016/j.molp.2016.08.003 27592586

[B44] Li H. Yue Y. Ding W. Chen G. Li L. Li Y.. (2020a). Genome-wide identification, classification, and expression profiling reveals *R2R3-MYB* transcription factors related to monoterpenoid biosynthesis in *Osmanthus fragrans* . Genes 11 (4), 353. doi: 10.3390/genes11040353 32224874 PMC7230838

[B45] Luo Y. Huang X. Song X. Wen B. Xie N. Wang K.. (2022). Identification of a *WRKY* transcriptional activator from *Camellia sinensis* that regulates methylated EGCG biosynthesis. Horticult Res. 2022 (9), uhac024. doi: 10.1093/hr/uhac024 PMC9071374 35184160

[B46] Lu S. Wang J. Chitsaz F. Derbyshire M. K. Geer R. C. Gonzales N. R.. (2020). CDD/SPARCLE: the conserved domain database in 2020. Nucleic Acids Res. 48 (D1), D265–D268. doi: 10.1093/nar/gkz991 31777944 PMC6943070

[B47] Mao P. Jin X. Bao Q. Mei C. Zhou Q. Min X.. (2020). *WRKY* transcription factors in *Medicago sativa* l.: genome-wide identification and expression analysis under abiotic stress. DNA Cell Biol. 39 (12), 2212–2225. doi: 10.1089/dna.2020.5726 33156699

[B48] Mistry J. Chuguransky S. Williams L. Qureshi M. Salazar G. A. Sonnhammer E. L.. (2021). Pfam: The protein families database in 2021. Nucleic Acids Res. 49 (D1), D412–D419. doi: 10.1093/nar/gkaa913 33125078 PMC7779014

[B49] Moore R. C. Purugganan M. D. (2003). The early stages of duplicate gene evolution. Proc. Natl. Acad. Sci. 100 (26), 15682–15687. doi: 10.1073/pnas.2535513100 14671323 PMC307628

[B50] Naoumkina M. A. He X. Z. Dixon R. A. (2008). Elicitor-induced transcription factors for metabolic reprogramming of secondary metabolism in *Medicago truncatula* . BMC Plant Biol. 8 (1), 132. doi: 10.1186/1471-2229-8-132 19102779 PMC2628384

[B51] Nicholas K. B. (1997). GeneDoc: analysis and visualization of genetic variation. Embnew. News 4, 14.

[B52] Ning P. Liu C. Kang J. Lv J. (2017). Genome-wide analysis of *WRKY* transcription factors in wheat (*Triticum aestivum* l.) and differential expression under water deficit condition. PeerJ 5, e3232. doi: 10.7717/peerj.3232 28484671 PMC5420200

[B53] Ohme-Takagi M. Suzuki K. Shinshi H. (2000). Regulation of ethylene-induced transcription of defense genes. Plant Cell Physiol. 41 (11), 1187–1192. doi: 10.1093/pcp/pcd057 11092902

[B54] Olive M. R. Walker J. C. Singh K. Ellis J. G. Llewellyn D. Peacock W. J.. (1991). The anaerobic responsive element. Plant Mol. Biol. 212 (1), 673–684. doi: 10.1007/978-1-4615-3304-7_67

[B55] Patra B. Schluttenhofer C. Wu Y. Pattanaik S. Yuan L. (2013). Transcriptional regulation of secondary metabolite biosynthesis in plants. Biochim. Biophys. Acta (BBA)-Gene Regul. Mech. 1829 (11), 1236–1247. doi: 10.1016/j.bbagrm.2013.09.006 24113224

[B56] Peng W. Liu Y. Hu M. Zhang M. Yang J. Liang F.. (2019). *Toona sinensis*: a comprehensive review on its traditional usages, phytochemisty, pharmacology and toxicology. Rev. Bras. Farmacognosia 29 (1), 111–124. doi: 10.1016/j.bjp.2018.07.009 PMC7103134 32287507

[B57] Ren L. Wan W. Xie Y. Chen B. Wang G. Yin D.. (2021). Identification of volatile aroma compounds of *Toona sinensis* (a. juss) roem buds and investigation of genes expression profiles confering aroma production. Pak. J. Bot. 53 (4), 1459–1464. doi: 10.30848/Pjb2021-4(32

[B58] Rinerson C. I. Rabara R. C. Tripathi P. Shen Q. J. Rushton P. J. (2015). The evolution of *WRKY* transcription factors. BMC Plant Biol. 15 (1), 1–18. doi: 10.1186/s12870-015-0456-y 25849216 PMC4350883

[B59] Rosado D. Ackermann A. Spassibojko O. Rossi M. Pedmale U. V. (2022). *WRKY* transcription factors and ethylene signaling modify root growth during the shade-avoidance response. Plant Physiol. 188 (2), 1294–1311. doi: 10.1093/plphys/kiab493 34718759 PMC8825332

[B60] Ross C. A. Liu Y. Shen Q. J. (2007). The *WRKY* gene family in rice (*Oryza sativa*). J. Integr. Plant Biol. 49 (6), 827–842. doi: 10.1111/j.1744-7909.2007.00504.x

[B61] Rushton P. J. Somssich I. E. Ringler P. Shen Q. J. (2010). *WRKY* transcription factors. Trends Plant Sci. 15 (5), 247–258. doi: 10.1016/j.tplants.2010.02.006 20304701

[B62] Schluttenhofer C. Yuan L. (2015). Regulation of specialized metabolism by *WRKY* transcription factors. Plant Physiol. 167 (2), 295–306. doi: 10.1104/pp.114.251769 25501946 PMC4326757

[B63] Song A. Li P. Jiang J. Chen S. Li H. Zeng J.. (2014). Phylogenetic and transcription analysis of chrysanthemum *WRKY* transcription factors. Int. J. Mol. Sci. 15 (8), 14442–14455. doi: 10.3390/ijms150814442 25196345 PMC4159861

[B64] Spyropoulou E. A. Haring M. A. Schuurink R. C. (2014). RNA Sequencing on *Solanum lycopersicum* trichomes identifies transcription factors that activate terpene synthase promoters. BMC Genomics 15 (1), 1–16. doi: 10.1186/1471-2164-15-402 24884371 PMC4041997

[B65] Sui J. Qu C. Yang J. Zhang W. Ji Y. (2019). Transcriptome changes in the phenylpropanoid pathway in senescing leaves of *Toona sinensis* . Acta Physiologiae Plantarum 41 (7), 1–15. doi: 10.1007/s11738-019-2915-9 PMC7088779 32214546

[B66] Sun Y. Niu Y. Xu J. Li Y. Luo H. Zhu Y.. (2013). Discovery of *WRKY* transcription factors through transcriptome analysis and characterization of a novel methyl jasmonate-inducible *PqWRKY1* gene from *Panax quinquefolius* . Plant Cell Tissue Organ Culture 114 (2), 269–277. doi: 10.1007/s11240-013-0323-1

[B67] Suttipanta N. Pattanaik S. Kulshrestha M. Patra B. Singh S. K. Yuan L. (2011). The transcription factor *CrWRKY1* positively regulates the terpenoid indole alkaloid biosynthesis in *Catharanthus roseus* . Plant Physiol. 157 (4), 2081–2093. doi: 10.1104/pp.111.181834 21988879 PMC3327198

[B68] Thompson J. D. Gibson T. J. Higgins D. G. (2003). Multiple sequence alignment using ClustalW and ClustalX. Curr. Protoc. Bioinf. 1), 2.3. doi: 10.1002/0471250953.bi0203s00 18792934

[B69] Wang Y. Chen F. Ma Y. Zhang T. Sun P. Lan M.. (2021). An ancient whole-genome duplication event and its contribution to flavor compounds in the tea plant (*Camellia sinensis*). Horticult Res. 2021 (8), 176–188. doi: 10.1038/s41438-021-00613-z PMC8325681 34333548

[B70] Wang Y. Tang H. DeBarry J. D. Tan X. Li J. Wang X.. (2012). MCScanX: a toolkit for detection and evolutionary analysis of gene synteny and collinearity. Nucleic Acids Res. 40 (7), e49–e49. doi: 10.1093/nar/gkr1293 22217600 PMC3326336

[B71] Wang Q. Wang M. Zhang X. Hao B. Kaushik S. Pan Y. (2011). *WRKY* gene family evolution in *Arabidopsis thaliana* . Genetica 139 (8), 973–983. doi: 10.1007/s10709-011-9599-4 21805321

[B72] Wang P. Yue C. Chen D. Zheng Y. Zhang Q. Yang J.. (2019). Genome-wide identification of *WRKY* family genes and their response to abiotic stresses in tea plant (*Camellia sinensis*). Genes Genomics 41 (1), 17–33. doi: 10.1007/s13258-018-0734-9 30238224

[B73] Wang D. Zhang Y. Zhang Z. Zhu J. Yu J. (2010). KaKs_Calculator 2.0: a toolkit incorporating gamma-series methods and sliding window strategies. Genomics Proteomics Bioinf. 8 (1), 77–80. doi: 10.1016/S1672-0229(10)60008-3 PMC5054116 20451164

[B74] Wei K. Chen J. Chen Y. Wu L. Xie D. (2012). Molecular phylogenetic and expression analysis of the complete *WRKY* transcription factor family in maize. DNA Res. 19 (2), 153–164. doi: 10.1093/dnares/dsr048 22279089 PMC3325079

[B75] Wu Z. Li X. Liu Z. Li H. Wang Y. Zhuang J. (2016). Transcriptome-wide identification of *Camellia sinensis WRKY* transcription factors in response to temperature stress. Mol. Genet. Genomics 291 (1), 255–269. doi: 10.1007/s00438-015-1107-6 26308611

[B76] Xiao Y. Zhou L. Lei X. Cao H. Wang Y. Dou Y.. (2017). Genome-wide identification of *WRKY* genes and their expression profiles under different abiotic stresses in *Elaeis guineensis* . PloS One 12 (12), e0189224. doi: 10.1371/journal.pone.0189224 29228032 PMC5724828

[B77] Xie L. Yan T. Li L. Chen M. Ma Y. Hao X.. (2021). The *WRKY* transcription factor *AaGSW2* promotes glandular trichome initiation in *Artemisia annua* . J. Exp. Bot. 72 (5), 1691–1701. doi: 10.1093/jxb/eraa523 33165526

[B78] Xu Y. H. Wang J. W. Wang S. Wang J. Chen X. Y. (2004). Characterization of *GaWRKY1*, a cotton transcription factor that regulates the sesquiterpene synthase gene (+)-δ-cadinene synthase-a. Plant Physiol. 135 (1), 507–515. doi: 10.1104/pp.104.038612 15133151 PMC429402

[B79] Yang C. Fang X. Wu X. Mao Y. Wang L. Chen X. (2012). Transcriptional regulation of plant secondary metabolism. J. Integr. Plant Biol. 54 (10), 703–712. doi: 10.1111/j.1744-7909.2012.01161.x 22947222

[B80] Yang Y. Ma Y. Yang S. Yue X. Peng W. (2020a). Chemical components analysis of *Toona sinensis* bark and wood by pyrolisis-gas chromatography-mass spectrometry. Asia-Pacific J. Chem. Eng. 15, e2487. doi: 10.1002/apj.2487

[B81] Yang Y. Wang N. Zhao S. (2020b). Functional characterization of a *WRKY* family gene involved in somatic embryogenesis in *Panax ginseng* . Protoplasma 257 (2), 449–458. doi: 10.1007/s00709-019-01455-2 31760482

[B82] Yang Y. Zhou Y. Chi Y. Fan B. Chen Z. (2017). Characterization of soybean *WRKY* gene family and identification of soybean *WRKY* genes that promote resistance to soybean cyst nematode. Sci. Rep. 7 (1), 1–13. doi: 10.1038/s41598-017-18235-8 29259331 PMC5736691

[B83] Yan H. Jia H. Chen X. Hao L. An H. Guo X. (2014). The cotton *WRKY* transcription factor *GhWRKY17* functions in drought and salt stress in transgenic *Nicotiana benthamiana* through ABA signaling and the modulation of reactive oxygen species production. Plant Cell Physiol. 55 (12), 2060–2076. doi: 10.1093/pcp/pcu133 25261532

[B84] Yu J. Wang J. Lin W. Li S. Li H. Zhou J.. (2005). The genomes of *Oryza sativa*: a history of duplications. PloS Biol. 3 (2), e38. doi: 10.1371/journal.pbio.0030038 15685292 PMC546038

[B85] Zhai X. Granvogl M. (2019). Characterization of the key aroma compounds in two differently dried *Toona sinensis* (A. juss.) roem. by means of the molecular sensory science concept. J. Agric. Food Chem. 67 (35), 9885–9894. doi: 10.1021/acs.jafc.8b06656 31090412

[B86] Zhang Y. Wang L. (2005). The *WRKY* transcription factor superfamily: its origin in eukaryotes and expansion in plants. BMC Evolutionary Biol. 5 (1), 1–12. doi: 10.1186/1471-2148-5-1 PMC544883 15629062

[B87] Zhao Q. Zhong X. Zhu S. Wang K. Tan G. Meng P.. (2022). Research advances in *Toona sinensis*, a traditional chinese medicinal plant and popular vegetable in China. Diversity 14 (7), 572. doi: 10.3390/d14070572

[B88] Zhou C. Lin Q. Lan J. Zhang T. Liu X. Miao R.. (2020). *WRKY* transcription factor *OsWRKY29* represses seed dormancy in rice by weakening abscisic acid response. Front. Plant Sci. 11. doi: 10.3389/fpls.2020.00691 PMC7268104 32536934

