# Supplementary figures and images for: Genome-wide analysis of WRKY transcription factor genes in Toona sinensis: An insight into evolutionary characteristics and terpene synthesis
Source: Front Plant Sci. 2023 Jan 20;13:1063850. doi: 10.3389/fpls.2022.1063850 (PMC9895799; doi:10.3389/fpls.2022.1063850)

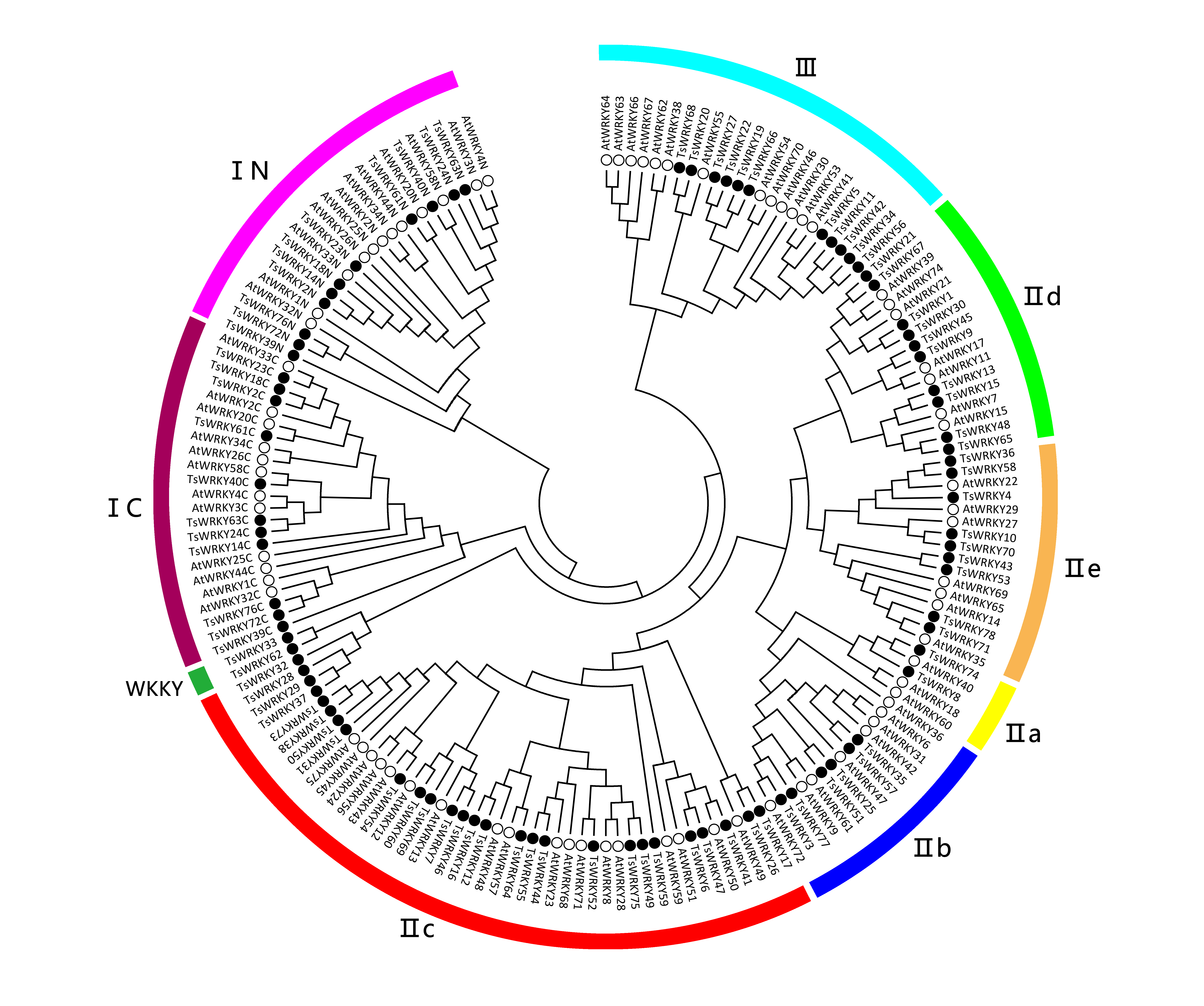

Supplement: Supplementary Figure 1 — A phylogenetic tree of WRKY domains from T. sinensis and A. thaliana. [file Image_1.jpeg]

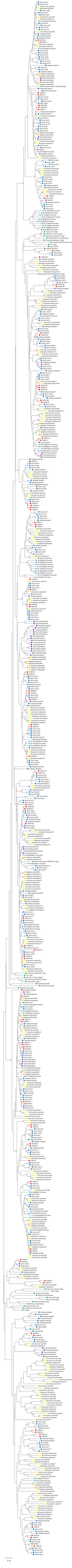

Supplement: Supplementary Figure 2 — A phylogenetic tree of WRKY domains from T. sinensis and other species in the green lineage. [file Image_2.jpeg]
